# Supplementary material for: Respiratory diseases in survivors of adult cancer compared with the general population: a systematic review protocol
Source: BMJ Open. 2022 Nov 21;12(11):e066029. doi: 10.1136/bmjopen-2022-066029 (PMC9680143; doi:10.1136/bmjopen-2022-066029)
Supplement: Author's manuscript [file bmjopen-2022-066029.draft_revisions.pdf]

BMJ Open is committed to open peer review. As part of this commitment we make the peer review history of every article we publish publicly available.

When an article is published we post the peer reviewers' comments and the authors' responses online. We also post the versions of the paper that were used during peer review. These are the versions that the peer review comments apply to.

The versions of the paper that follow are the versions that were submitted during the peer review process. They are not the versions of record or the final published versions. They should not be cited or distributed as the published version of this manuscript.

BMJ Open is an open access journal and the full, final, typeset and author-corrected version of record of the manuscript is available on our site with no access controls, subscription charges or pay-per-view fees (http://bmjopen.bmj.com).

If you have any questions on BMJ Open's open peer review process please email info.bmjopen@bmj.com

BMJ Open

Respiratory diseases in cancer survivors compared with the general population: a systematic review protocol.

Journal:	*BMJ Open*
Manuscript ID	bmjopen-2022-066029
Article Type:	Protocol
Date Submitted by the Author:	27-Jun-2022
Complete List of Authors:	Andresen, Kirsty; London School of Hygiene & Tropical Medicine, Non Communicable Disease Epidemiology<br>Carreira, Helena; London School of Hygiene and Tropical Medicine Faculty of Epidemiology and Population Health, Non-communicable diseases epidemiology<br>Quint, Jennifer; Imperial College London, NHLI; Imperial College London<br>Bhaskaran, Krishnan; LSHTM, NCDE
Keywords:	ONCOLOGY, Epidemiology < ONCOLOGY, EPIDEMIOLOGY, Respiratory infections < THORACIC MEDICINE, Asthma < THORACIC MEDICINE, Chronic airways disease < THORACIC MEDICINE

SCHOLARONE™
Manuscripts

Respiratory diseases in cancer survivors compared with the general population: a systematic review protocol.

Kirsty Andresen[1], Helena Carreira[1], Jennifer K Quint[2], Krishnan Bhaskaran[1]

[1]Department of Non-Communicable Disease Epidemiology, Faculty of Epidemiology and Population Health, London School of Hygiene & Tropical Medicine, Keppel Street, London, WC1E 7HT UK

[2]National Heart and Lung Institute, Imperial College London

Contributor information

Kirsty Andresen, e-mail: kirsty.andresen1@lshtm.ac.uk (corresponding author)

Helena Carreira, e-mail: Helena.carreira@lshtm.ac.uk

Jennifer K Quint, e-mail: j.quint@imperial.ac.uk

Krishnan Bhaskaran, e-mail: Krishnan.bhaskaran@lshtm.ac.uk

Word count: 3,648

For peer review only - http://bmjopen.bmj.com/site/about/guidelines.xhtml

1 Abstract (232 words)

Introduction: There is concern that cancer survivors may be at increased risk of respiratory infections and of exacerbations of pre-existing respiratory conditions. Considering the high prevalence of respiratory disease in the general population, increased respiratory disease risk in cancer survivors could translate into an important impact on morbidity and mortality. The aim of this systematic review is to summarise and assess the quality of all studies comparing respiratory outcomes between cancer survivors and individuals with no history of cancer.

Methods and analysis: This systematic literature review will be conducted using Medline and EMBASE. We will include cohort or case-control studies that provide a comparative estimate of the risk of a respiratory disease of interest against a cohort of cancer-free individuals. No geographic, time or language restrictions will be applied. We will assess the risk of bias using the Scottish Intercollegiate Guidelines Network (SIGN) methodology checklists. Results will be summarised by outcome, cancer type and cancer survivorship definition. If sufficient numbers of homogeneous studies are found, summary measures of association will be calculated using random effects meta-analysis models.

Ethics and dissemination: Ethical approval is not applicable to our study. The results will be used to identify evidence gaps and priorities for future research to understand respiratory morbidity in cancer survivors and identify possible mitigation strategies. Results from this review will be disseminated to clinical audiences and submitted to a peer-reviewed journal when completed.

2 Strengths and limitations

- To our knowledge, this will be the first study to systematically report a comprehensive picture of the spectrum of respiratory conditions that affect cancer survivors as well as understand if cancer-specific or respiratory condition-specific associations have been noted in the literature.

For peer review only - http://bmjopen.bmj.com/site/about/guidelines.xhtml

- Studies comparing long-term outcomes in cancer survivors and controls may be subject to a number of biases including selection bias, detection biases, and competing-risks. However, the risk of bias will be assessed using Scottish Intercollegiate Guidelines Network (SIGN) methodology checklists.
- Though no geographic, time or language restrictions will be applied, we may miss relevant articles not captured by English language search engines.

3 Keywords

Cancer survivors, respiratory disorders, Covid-19, pneumonia, influenza, respiratory tract infections, asthma, COPD, interstitial lung disease, systematic review.

4 Introduction

Currently, 50% of people diagnosed with cancer are expected to survive for 10 or more years (1). The proportion is likely to increase further considering the rapid and continual improvements in cancer management and treatment. As more patients survive longer, they may experience additional health challenges during their survivorship. There is evidence that cancer survivors are at an increased risk of developing certain adverse outcomes, such as cardiovascular diseases (2) and mental health issues (3), that may cause morbidity and impaired quality of life. However, the long-term respiratory consequences of cancer and its treatment are less clear.

Respiratory diseases have an immense worldwide burden of morbidity and mortality, accounting for more than 10% of all disability-adjusted life-years (DALYs), second only to cardiovascular diseases (including stroke) (4). It is thought that the effects of cancer and its treatment may increase the risk of respiratory infections due to weakened immune systems (5). Furthermore, a quarter of cancer patients have been diagnosed with a pre-existing respiratory comorbidities (6) and there is concern that cancer and its treatment or shared risk factors may be conducive to worse outcomes than in the general population (7). Considering the high prevalence of respiratory disease in the general population, even a small increase in respiratory disease risk in cancer survivors will translate into an

For peer review only

For peer review only - http://bmjopen.bmj.com/site/about/guidelines.xhtml

important impact on morbidity and mortality. Thus, identifying possible strategies to mitigate respiratory morbidity in cancer survivors would suppose an important improvement in absolute terms.

We aim to conduct a systematic review to summarise and assess the quality of the current knowledge from studies comparing cancer survivors to individuals with no history of cancer for respiratory outcomes. We aim to evaluate relative risks of a broad spectrum of respiratory outcomes across a wide range of cancer types, in order to build a complete picture of the evidence on respiratory health during cancer survivorship.

Methods and analysis

This study protocol has been designed according to the Preferred Reporting Items for Systematic Review and Meta-Analysis Protocols (PRISMA-P), registered in the International Prospective Register of Ongoing Systematic Reviews (PROSPERO) and will be reported according to the PRISMA reporting guidelines. PROSPERO registration number: CRD42022311557.

4.1 Objectives

The specific objectives of this study are:

1. Summarise the current evidence on the association between cancer survivorship and subsequent respiratory disease.
2. Critically appraise the quality of the available evidence and evaluate the main sources of bias.
3. Identify evidence gaps and priorities for future research on respiratory health in cancer survivors.

For peer review only

For peer review only - http://bmjopen.bmj.com/site/about/guidelines.xhtml

4.2 Search strategy

The search will include articles published in Medline and Embase through to 08 March 2022. No geographic, time or language restrictions will be applied. We will use OVID© search interface to simultaneously search both of the selected databases. The coverage of EMBASE spans from 1974 and Medline from 1946. We do not plan to include grey literature as epidemiological studies providing measures of effect with enough information to be assessable are likely to be published in academic journals. Furthermore, we do not plan to routinely contact all study authors. We will search title, abstract and subject headings using our created search expression. The search expression will include keywords and subject headings (both MeSH and Emtree) for the target population (cancer survivors) and the target outcomes (respiratory diseases). As an additional step to identify studies that may have been missed by the initial search strategy, we will examine the reference lists of all studies identified in the initial database search that meet the selection criteria. The list of selected respiratory outcomes and associated search terms were confirmed by a respiratory consultant (JQ). The search expression is included in Table 1

*Table 1. Search expression for both MEDLINE and EMBASE**

**Search expression**
1 *Cancer Survivors/ or exp cancer survivor
2 (cancer survivor* or "living with and beyond cancer" or "living with cancer").mp.
3 (respiratory and (outcome* or disease* or infection* or condition* or disorder or manifestation*)).mp.
4 (lung and (outcome* or disease* or infection* or condition* or disorder or manifestation*)).mp.
5 (pulmonary and (outcome* or disease* or infection* or condition* or disorder or manifestation*)).mp.
6 exp respiratory tract disease/ or exp lung disease/
7 ("upper respiratory infection*" or "upper respiratory tract infection*" or nasopharyngitis or rhinitis or pharyngitis or sinusitis or tonsilitis or laryngitis or tracheitis or laryngotracheitis).mp.
8 respiratory tract infections/ or common cold/ or empyema, pleural/ or laryngitis/ or pharyngitis/ or rhinitis/ or sinusitis/ or whooping cough/ or exp upper respiratory tract/ or exp upper respiratory tract congestion/ or exp upper respiratory tract infection/
9 bronchitis.mp. or Bronchitis/ or Infectious bronchitis virus/
10 pulmonary diseases/ or chronic obstructive/ or bronchitis, chronic/ or pulmonary emphysema/ or Bronchitis, Chronic/ or exp chronic bronchitis/ or exp bronchitis/
11 (influenza* or flu).mp. or influenza, human/

For peer review only

For peer review only - http://bmjopen.bmj.com/site/about/guidelines.xhtml

12	exp influenza/
13	Pneumonia/ or pneumonia.mp.
14	exp pneumonia/
15	Asthma/ or asthma.mp.
16	exp asthma/
17	(chronic obstructive pulmonary disease or COPD or chronic bronchitis or emphysema).mp.
18	exp chronic obstructive lung disease/
19	interstitial lungs disease.mp. or Lung Diseases, Interstitial/
20	exp interstitial lung disease/
21	Pulmonary Fibrosis/
22	(pulmonary fibrosis or lung fibrosis).mp.
23	exp lung fibrosis/
24	pneumonitis.mp.
25	exp coronavirus disease 2019/ or exp COVID-19/ or (Covid-19 or SARS-CoV-2 or coronavirus 19).mp.
26	3 or 4 or 5 or 6 or 7 or 8 or 9 or 10 or 11 or 12 or 13 or 14 or 15 or 16 or 17 or 18 or 19 or 20 or 21 or 22 or 23 or 24 or 25
27	1 or 2
32	26 and 27

*Both MeSH terms and Emtree terms were included in our search expression.

We defined respiratory disease as any upper or lower respiratory condition. See Table 2 for list of included respiratory diseases.

Table 2. Categorisation of respiratory diseases included in the systematic literature review

**Category**	**Conditions of interest**
Covid-19	Infection with SARS-CoV-19
Upper respiratory infections	Nasopharyngitis, rhinitis, pharyngitis, sinusitis, tonsilitis, laryngitis, tracheitis, laryngotracheitis
Bronchitis	Acute Bronchitis
Influenza	Influenza
Pneumonia	Pneumonia
Asthma	Asthma
COPD	chronic obstructive pulmonary disease, chronic bronchitis, emphysema
Pneumonitis	pneumonitis
Fibrosis	pulmonary fibrosis
Interstitial lung disease	Interstitial lung disease

4.3 Screening

The screening will be conducted in two stages:

For peer review only - http://bmjopen.bmj.com/site/about/guidelines.xhtml

1) Screening of titles and abstracts: Titles and abstracts will be screened against the selection criteria by one reviewer. A second reviewer will screen a random sample of ten percent of articles identified in the initial search. Any discrepancies will be resolved by discussion between the reviewers and the wider study team. Studies which meet all the inclusion criteria and none of the exclusion criteria (see below) will be considered for full text review. If the study cannot be unequivocally excluded during this phase, it will be considered for full text review.
2) Full text review: This step will be conducted independently by two reviewers in full. Any discrepancies will be resolved by discussion between the reviewers and the wider study team.

We will use the reference manager Endnote 20 to store citations and screen all identified references.

4.4 Selection criteria

The inclusion criteria for the systematic review will be:

1) The exposure is cancer survivorship. In this review our focus will be on the survivorship period after initial active cancer treatment, so the exposed group must include follow-up time beyond the period of active treatment or the first year since diagnosis.
2) The study evaluates at least one respiratory disease of interest as an outcome of interest (See Table 2 for details).
3) The study involves a comparator cohort of cancer free individuals. No other criteria will be set for the comparator.
4) The study has an observational longitudinal cohort, matched cohort or case-control design.
5) The study provides a relative measure of effect for a respiratory disease of interest in individuals with cancer compared to cancer-free controls. Examples of relevant measures of effect are relative risk ratios, odds ratios, rate ratios, and hazard ratios. If studies provide

For peer review only - http://bmjopen.bmj.com/site/about/guidelines.xhtml

For peer review only

sufficient information on measures of frequency for any of the respiratory diseases of interest measured in individuals with cancer and cancer-free controls to allow the calculation measure of effect between the groups, the study will also be included.

We will exclude:

1) Duplicate studies, defined as repeated articles, using the remove duplicate function of the software prior to conducting the manual screening. Any articles not identified by the software, will be removed manually by the reviewers.
2) Studies where the exposed group is exclusively composed of cancer patients under active treatment as well as cancer cohorts whose average follow-up time is ≤1 year.
3) Studies in which the exposed group includes individuals without cancer (e.g., families and caregivers of the patient).
4) Intervention studies, cross-sectional studies and studies that do not use original data (such as review articles, comments, letters, editorials and protocols). Any review study will be excluded but flagged and the citation lists will be evaluated in order to identify potentially eligible studies that were not captured in our search.
5) Conference abstracts will be excluded if no corresponding full-text journal article is found as we consider that the information in an abstract will not be sufficient to complete our data extraction nor conduct an accurate bias assessment.

While articles will not be excluded on the basis of language, practical limitations mean that we will only evaluate those studies captured by an English search strategy. Articles in languages not spoken by the study team will be translated using translation software. We will exclude full-text articles which are written in non-roman alphabets due to the limitations of our translation software.

Reasons for exclusion will be collected in a cascade manner, meaning that papers that fail to meet any of the selection criteria will be automatically excluded and not further assessed. Where multiple papers are found to report data conducted on the same study population sample, these will be

For peer review only - http://bmjopen.bmj.com/site/about/guidelines.xhtml

flagged, but not excluded. The results of these studies cannot be considered independent and thus the inclusion and/or interpretation of these studies will be considered on a case-by-case basis after discussion within the study team. Any inclusion or exclusions of studies of this type will be detailed in the data extraction phase and reported in the results section of the report.

4.5 Data extraction

Data will initially be extracted from included studies into a spreadsheet. Data extraction will commence in July2022. At minimum, we will detail information on the study article, population sample description, study design, exposure, comparator and outcome definitions, follow-up, measures of effect, measures of frequency and statistical methodology. The exact data items extracted are listed in Table 3.

Table 3. Data items for extraction

Data items:
Administrative:
• Authors<br>• Journal<br>• Year of publication<br>• Financial/funding sources<br>• Conflict of interest reported
Data/design/population:
• Country/countries of origin<br>• Data source<br>• Time period covered<br>• Study setting<br>• Study design<br>• Sample size
Comparison group:
• Selection criteria of the comparison group<br>• Mean age of the comparison group
Exposure:
• Selection criteria for exposed group<br>• Definition of cancer survivorship<br>• Time since cancer diagnosis (summary stats)<br>• Cancer Stage (summary stats)
Outcome:

For peer review only

For peer review only - http://bmjopen.bmj.com/site/about/guidelines.xhtml

• Respiratory disease category<br>• Respiratory disease definition<br>• Duration of follow-up (if applicable)
Statistical methods:
• Statistical method for the calculation of estimates<br>• Statistical method for the calculation of the method of precision<br>• Confounders (if applicable)
Results
• Measure of frequency + precision estimate (comparison group)<br>• Measure of frequency + precision estimate (exposed group)<br>• Measure of effect + precision estimate (crude)<br>• Measure of effect + precision estimate (adjusted)

When multiple *different* respiratory disease conditions or cancer types are reported in the same study, we will record or calculate information for all cancer types and respiratory outcomes. When multiple estimates are reported for the *same condition* (e.g., if probable and confirmed influenza were reported as separate outcomes) we will only record or calculate the measure of effect for the outcome corresponding to the stronger certainty of diagnosis (in our illustrative example, we would select confirmed influenza). If the results are stratified by severity, we will record all severity categories for said outcome.

4.6 Risk of bias assessment

We will use the Scottish Intercollegiate Guidelines Network (SIGN) methodology checklists as guidance to assess the risk of bias for each study design. The checklists are provided in the appendix (Table A1 and Table A2). In brief, we will assess if the study has a focussed aim, and whether there were opportunities for bias in in the definition of the exposures and outcomes, selection of the study population and comparator group, and potential differential losses during follow up. We will also assess how each study addresses confounding.

4.7 Data Analysis

We will initially present the key characteristics and results of all studies in descriptive tables. We will stratify the presentation of results by likely sources of heterogeneity such as cancer type and

For peer review only - http://bmjopen.bmj.com/site/about/guidelines.xhtml

respiratory disease category (See Table 2). If sufficient studies are found, we will also stratify by different population definitions (i.e., if we find multiple definitions for cancer survivorship). We will meta-analyse groups of studies if we find sufficient studies that are sufficiently homogeneous in terms of population and outcomes for which the calculation of a summary measure would be meaningful. In the case that a summary estimate is calculated, we will use random-effects model. We will explore the consistency between studies by using the I^2 statistic.

4.8 Patient and public involvement

No patients were involved in the development of this protocol

5 Ethics and dissemination

To our knowledge, no comparable review has previously been conducted on respiratory outcomes in survivors of adult cancer. We aim to identify all available literature on a wide range of respiratory outcomes and cancer types considered, with no language, geographical or date limitations. This will provide a comprehensive picture of the spectrum of respiratory conditions that affect cancer survivors as well as understand if cancer-specific or respiratory condition-specific associations have been noted in the literature. Considering the high prevalence of respiratory disease in the general population, even a small increase in respiratory disease risk in cancer survivors will translate into an important impact on morbidity and mortality. The results of this review can be used to better understand respiratory morbidity in cancer survivors, identify evidence gaps and priorities for future research, and help devise possible mitigation strategies. Results will be disseminated to clinical audiences and may support clinicians and decision-makers in developing survivorship care guidelines.

While this protocol has been developed according to the PRISMA-P guidelines and has been registered on PROSPERO (ref: PROSPERO CRD42022311557), some potential limitations are worth noting. Firstly, there is currently no universally accepted definition of cancer survivorship (8), and

For peer review only - http://bmjopen.bmj.com/site/about/guidelines.xhtml

this review focusses on the late respiratory challenges of cancer and its treatment. Consequentially, we have adopted the European Organisation for Research and Treatment of Cancer (EORTC) definition which defines cancer survivor as “individuals diagnosed with cancer who have completed their primary treatment (maintenance treatment can be ongoing)”(9). We have used an inclusive approach towards this definition and will consider that the period of active treatment is expected to have been completed after 1 year since diagnosis, thus we will not exclude studies that include patients from cancer diagnosis with a mean follow-up of >1 year. This is in line with the International Agency for Research on Cancer (IARC) categorisation of net survival: 1 year survival (initial treatment), 3 year survival (clinical follow-up) and five year survival (cure) (10).

Studies comparing long-term outcomes in cancer survivors and controls may be subject to a number of biases including selection bias, detection biases, and competing-risks. We will evaluate the risk of bias in each included study in order to aid the interpretation of results, though we acknowledge that not all types of bias are specifically addressed by the SIGN checklists. Our review will also have other limitations. We may miss some relevant studies due to an imperfect search strategy, though hand searching of reference lists should identify additional papers and allow us to refine our search terms if needed. Finally, we are not including conference abstracts or preprints which could potentially include some relevant results.

Considering the high prevalence of respiratory disease in the general population, even a small increase in respiratory disease risk in cancer survivors will translate into an important impact on morbidity and mortality. The results of this review can be used to better understand respiratory morbidity in cancer survivors, identify evidence gaps and priorities for future research, and help devise possible mitigation strategies.

For peer review only - http://bmjopen.bmj.com/site/about/guidelines.xhtml

6 Abbreviation list

EORTC	European Organisation for Research and Treatment of Cancer
IARC	International Agency for Research on Cancer
PRISMA	Preferred reporting items for systematic review and meta-analysis
PRISMA – P	Preferred reporting items for systematic review and meta-analysis protocol
PROSPERO	International Prospective Register of Ongoing Systematic Reviews
SIGN	Scottish Intercollegiate Guidelines Network

7 Conflict of interests

No competing interests to declare

8 Author Statement

KB, HC and KA designed the study. KA wrote the first draft of the protocol. JQ provided clinical input for the selection of the study outcomes. All authors made substantial contributions to the manuscript, revised it critically for important intellectual content and read and approved the final version. KA is the guarantor of the review.

9 Data statement

The protocol is registered on PROSPERO (registration number: CRD42022311557). The data extraction sheet will be provided as an appendix to any published article

10 Funding

KA is supported by the Medical Research Council Intercollegiate Doctoral Training Partnership Studentship (Grant no MR/N013638/1). KB is funded by a Wellcome Senior Research Fellowship (grant number 220283/z/20/z). The funders had no role in developing this protocol.

11 Acknowledgements

We thank Ruchika Jain for agreeing to assist in conducting the screening of full-text articles.

For peer review only - http://bmjopen.bmj.com/site/about/guidelines.xhtml

For peer review only

12 References

1. Cancer Research UK. Cancer survival statistics for all cancers combined [Available from: https://www.cancerresearchuk.org/health-professional/cancer-statistics/survival/all-cancers-combined.
2. Strongman H, Gadd S, Matthews A, Mansfield KE, Stanway S, Lyon AR, et al. Medium and long-term risks of specific cardiovascular diseases in survivors of 20 adult cancers: a population-based cohort study using multiple linked UK electronic health records databases. Lancet (London, England). 2019;394(10203):1041-54.
3. Carreira H, Williams R, Dempsey H, Stanway S, Smeeth L, Bhaskaran K. Quality of life and mental health in breast cancer survivors compared with non-cancer controls: a study of patient-reported outcomes in the United Kingdom. J Cancer Surviv. 2021;15(4):564-75.
4. Wang H, Naghavi M, Allen C, Barber RM, Bhutta ZA, Carter A, et al. Global, regional, and national life expectancy, all-cause mortality, and cause-specific mortality for 249 causes of death, 1980–2015: a systematic analysis for the Global Burden of Disease Study 2015. The Lancet. 2016;388(10053):1459-544.
5. Kang DH, Weaver MT, Park NJ, Smith B, McArdle T, Carpenter J. Significant impairment in immune recovery after cancer treatment. Nurs Res. 2009;58(2):105-14.

For peer review only - http://bmjopen.bmj.com/site/about/guidelines.xhtml

6. Ogle KS, Swanson GM, Woods N, Azzouz F. Cancer and comorbidity. Cancer. 2000;88(3):653-63.
7. Carreira H, Strongman H, dos-Santos-Silva I, Smeeth L, Bhaskaran K, Peppa M, et al. Prevalence of COVID-19-related risk factors and risk of severe influenza outcomes in cancer survivors: A matched cohort study using linked English electronic health records data. EClinicalMedicine. 2020;29-30:100656.
8. Dirven L, van de Poll-Franse LV, Aaronson NK, Reijneveld JC. Controversies in defining cancer survivorship. The Lancet Oncology. 2015;16(6):610-2.
9. Moser EC, Meunier F. Cancer survivorship: A positive side-effect of more successful cancer treatment. European Journal of Cancer Supplements. 2014;12(1):1-4.
10. Arnold M RM, Lam F, Bray F, Ervik M, Soerjomataram I (2019). . accessed [day/month/year]. ICBP SURVMARK-2 online tool: International Cancer Survival Benchmarking. Lyon, France: International Agency for Research on Cancer Available from: http://gco.iarc.fr/survival/survmark, 2019 [

For peer review only

For peer review only - http://bmjopen.bmj.com/site/about/guidelines.xhtml

Appendix

Table A 1. SIGN checklist for cohort studies

SIGN	**Methodology Checklist 3: Cohort studies**		
Study identification (*Include author, title, year of publication, journal title, pages*)			
Guideline topic:		Key Question No:	Reviewer:

Before completing this checklist, consider:

1. Is the paper really a cohort study? If in doubt, check the study design algorithm available from SIGN and make sure you have the correct checklist.
2. Is the paper relevant to key question? Analyse using PICO (Patient or Population Intervention Comparison Outcome). IF NO REJECT (give reason below). IF YES complete the checklist..

Reason for rejection: 1. Paper not relevant to key question ☐ 2. Other reason ☐ (please specify):

Please note that a retrospective study (ie a database or chart study) cannot be rated higher than +.

SECTION 1: INTERNAL VALIDITY

In a well conducted cohort study:		***Does this study do it?***	
1.1	The study addresses an appropriate and clearly focused question.[i]	Yes □<br>Can't say □	No □
SELECTION OF SUBJECTS			
1.2	The two groups being studied are selected from source populations that are comparable in all respects other than the factor under investigation.[ii]	Yes □<br>Can't say □	No □<br>Does not apply □
1.3	The study indicates how many of the people asked to take part did so, in each of the groups being studied.[iii]	Yes □	No □<br>Does not apply □
1.4	The likelihood that some eligible subjects might have the outcome at the time of enrolment is assessed and taken into account in the analysis.[iv]	Yes □<br>Can't say □	No □<br>Does not apply □
1.5	What percentage of individuals or clusters recruited into each arm of the study dropped out before the study was completed.[v]		
1.6	Comparison is made between full participants and those lost to follow up, by exposure status.[vi]	Yes □<br>Can't say □	No □<br>Does not apply □

For peer review only

Table A 2. SIGN checlist for case-control studies

SIGN	**Methodology Checklist 4: Case-control studies**	
Study identification (*Include author, title, year of publication, journal title, pages*)		
Guideline topic:	Key Question No:	Reviewer:

Before completing this checklist, consider:

1. Is the paper really a case-control study? If in doubt, check the study design algorithm available from SIGN and make sure you have the correct checklist.
2. Is the paper relevant to key question? Analyse using PICO (Patient or Population Intervention Comparison Outcome). IF NO REJECT (give reason below). IF YES complete the checklist.

Reason for rejection: Reason for rejection: 1. Paper not relevant to key question ☐ 2. Other reason ☐ (please specify):

SECTION 1: INTERNAL VALIDITY

	In an well conducted case control study:	***Does this study do it?***
1.1	The study addresses an appropriate and clearly focused question.[i]	Yes ☐ No ☐ Can't say ☐
SELECTION OF SUBJECTS		
1.2	The cases and controls are taken from comparable populations.[ii]	Yes ☐ No ☐ Can't say ☐
1.3	The same exclusion criteria are used for both cases and controls.[iii]	Yes ☐ No ☐ Can't say ☐
1.4	What percentage of each group (cases and controls) participated in the study?[iv]	Cases:<br>Controls:
1.5	Comparison is made between participants and non-participants to establish their similarities or differences.[v]	Yes ☐ No ☐ Can't say ☐
1.6	Cases are clearly defined and differentiated from controls.[vi]	Yes ☐ No ☐ Can't say ☐

For peer review only

PRISMA-P 2015 Checklist

This checklist has been adapted for use with systematic review protocol submissions to BioMed Central journals from Table 3 in Moher D et al: Preferred reporting items for systematic review and meta-analysis protocols (PRISMA-P) 2015 statement. *Systematic Reviews* 2015 **4**:1

An Editorial from the Editors-in-Chief of *Systematic Reviews* details why this checklist was adapted - **Moher D, Stewart L & Shekelle P:** Implementing PRISMA-P: recommendations for prospective authors. *Systematic Reviews* 2016 **5**:15

Section/topic	#	Checklist item	Information reported Yes	Information reported No	Line number(s)
**ADMINISTRATIVE INFORMATION**					
**Title**					
Identification	1a	Identify the report as a protocol of a systematic review	☒	☐	Line 1-2
Update	1b	If the protocol is for an update of a previous systematic review, identify as such	☐	☒	Not applicable
**Registration**	2	If registered, provide the name of the registry (e.g., PROSPERO) and registration number in the Abstract	☒	☐	Line 81
**Authors**					
Contact	3a	Provide name, institutional affiliation, and e-mail address of all protocol authors; provide physical mailing address of corresponding author	☒	☐	Line 4-13
Contributions	3b	Describe contributions of protocol authors and identify the guarantor of the review	☒	☐	Line 272
**Amendments**	4	If the protocol represents an amendment of a previously completed or published protocol, identify as such and list changes; otherwise, state plan for documenting important protocol amendments	☐	☒	Not applicable
**Support**					
Sources	5a	Indicate sources of financial or other support for the review	☒	☐	Line 280-283
Sponsor	5b	Provide name for the review funder and/or sponsor	☒	☐	Line 280-283
Role of sponsor/funder	5c	Describe roles of funder(s), sponsor(s), and/or institution(s), if any, in developing the protocol	☒	☐	Line 280-283
**INTRODUCTION**					
**Rationale**	6	Describe the rationale for the review in the context of what is already known	☒	☐	Line 53-73
**Objectives**	7	Provide an explicit statement of the question(s) the review will address with reference to	☒	☐	Line 72-76

BioMed Central The Open Access Publisher

For peer review only - http://bmjopen.bmj.com/site/about/guidelines.xhtml

Section/topic	#	Checklist item	Information reported Yes	Information reported No	Line number(s)
		participants, interventions, comparators, and outcomes (PICO)			
**METHODS**					
**Eligibility criteria**	8	Specify the study characteristics (e.g., PICO, study design, setting, time frame) and report characteristics (e.g., years considered, language, publication status) to be used as criteria for eligibility for the review	☒	☐	Line 123-152
**Information sources**	9	Describe all intended information sources (e.g., electronic databases, contact with study authors, trial registers, or other grey literature sources) with planned dates of coverage	☒	☐	Line 91-103
**Search strategy**	10	Present draft of search strategy to be used for at least one electronic database, including planned limits, such that it could be repeated	☒	☐	Line 104
STUDY RECORDS					
Data management	11a	Describe the mechanism(s) that will be used to manage records and data throughout the review	☒	☐	Line 121
Selection process	11b	State the process that will be used for selecting studies (e.g., two independent reviewers) through each phase of the review (i.e., screening, eligibility, and inclusion in meta-analysis)	☒	☐	Line 109 - 121
Data collection process	11c	Describe planned method of extracting data from reports (e.g., piloting forms, done independently, in duplicate), any processes for obtaining and confirming data from investigators	☒	☐	Line 165-185
**Data items**	12	List and define all variables for which data will be sought (e.g., PICO items, funding sources), any pre-planned data assumptions and simplifications	☒	☐	Line 170
**Outcomes and prioritization**	13	List and define all outcomes for which data will be sought, including prioritization of main and additional outcomes, with rationale	☒	☐	Line 108 & Line 172 - 178
**Risk of bias in individual studies**	14	Describe anticipated methods for assessing risk of bias of individual studies, including whether this will be done at the outcome or study level, or both; state how this information will be used in data synthesis	☒	☐	Line 180-185
DATA					
**Synthesis**	15a	Describe criteria under which study data will be quantitatively synthesized	☒	☐	Line 180-181
	15b	If data are appropriate for quantitative synthesis, describe planned summary measures, methods of handling data, and methods of combining data from studies, including any planned exploration of consistency (e.g., I^2, Kendall's tau)	☒	☐	Line 186 -194
	15c	Describe any proposed additional analyses (e.g., sensitivity or subgroup analyses, meta-regression)	☒	☐	Line 186 -194
	15d	If quantitative synthesis is not appropriate, describe the type of summary planned	☒	☐	Line 186 -194
**Meta-bias(es)**	16	Specify any planned assessment of meta-bias(es) (e.g., publication bias across studies, selective reporting within studies)	☐	☒	Not applicable

For peer review only - http://bmjopen.bmj.com/site/about/guidelines.xhtml

Section/topic	#	Checklist item	Information reported		Line number(s)
			Yes	No	
**Confidence in cumulative evidence**	17	Describe how the strength of the body of evidence will be assessed (e.g., GRADE)	☐	☒	Not applicable

For peer review only

BioMed Central The Open Access Publisher

BMJ Open

Respiratory diseases in adult cancer survivors compared with the general population: a systematic review protocol.

Journal:	*BMJ Open*
Manuscript ID	bmjopen-2022-066029.R1
Article Type:	Protocol
Date Submitted by the Author:	21-Sep-2022
Complete List of Authors:	Andresen, Kirsty; London School of Hygiene & Tropical Medicine, Non Communicable Disease Epidemiology<br>Carreira, Helena; London School of Hygiene and Tropical Medicine Faculty of Epidemiology and Population Health, Non-communicable diseases epidemiology<br>Quint, Jennifer; Imperial College London, NHLI; Imperial College London<br>Bhaskaran, Krishnan; LSHTM, NCDE
**Primary Subject Heading**:	Oncology
Secondary Subject Heading:	Respiratory medicine
Keywords:	ONCOLOGY, Epidemiology < ONCOLOGY, EPIDEMIOLOGY, Respiratory infections < THORACIC MEDICINE, Asthma < THORACIC MEDICINE, Chronic airways disease < THORACIC MEDICINE

SCHOLARONE™
Manuscripts

Respiratory diseases in adult cancer survivors compared with the general population: a systematic review protocol.

Kirsty Andresen[1], Helena Carreira[1], Jennifer K Quint[2], Krishnan Bhaskaran[1]

[1]Department of Non-Communicable Disease Epidemiology, Faculty of Epidemiology and Population Health, London School of Hygiene & Tropical Medicine, Keppel Street, London, WC1E 7HT UK

[2]National Heart and Lung Institute, Imperial College London

Contributor information

Kirsty Andresen, e-mail: kirsty.andresen1@lshtm.ac.uk (corresponding author)

Helena Carreira, e-mail: Helena.carreira@lshtm.ac.uk

Jennifer K Quint, e-mail: j.quint@imperial.ac.uk

Krishnan Bhaskaran, e-mail: Krishnan.bhaskaran@lshtm.ac.uk

For peer review only

1 Abstract (232 words)

Introduction: There is concern that survivors of adult cancers may be at increased risk of respiratory infections and of exacerbations of pre-existing respiratory conditions. Considering the high

For peer review only - http://bmjopen.bmj.com/site/about/guidelines.xhtml

prevalence of respiratory disease in the general population, increased respiratory disease risk in survivors of adult cancers could translate into an important impact on morbidity and mortality. The aim of this systematic review is to summarise and assess the quality of all studies comparing respiratory outcomes between adult cancer survivors and individuals with no history of cancer.

Methods and analysis: This systematic literature review will be conducted using Medline, EMBASE and Cochrane. We will include cohort or case-control studies that provide a comparative estimate of the risk of a respiratory disease of interest in adult cancer survivors against a comparator cohort of cancer-free individuals. No geographic, time or language restrictions will be applied. We will assess the risk of bias using the Scottish Intercollegiate Guidelines Network (SIGN) methodology checklists. Results will be summarised by type of respiratory outcome, cancer type and cancer survivorship definition. If sufficient numbers of homogeneous studies are found, summary measures of association will be calculated using random effects meta-analysis models.

Ethics and dissemination: Ethical approval is not applicable to our study. The results will be used to identify evidence gaps and priorities for future research to understand respiratory morbidity in cancer survivors and identify possible mitigation strategies. Results from this review will be disseminated to clinical audiences and submitted to a peer-reviewed journal when completed. This study has been registered on PROSPERO (registration number: CRD42022311557).

For peer review only

2 Strengths and limitations

- The search terms for respiratory outcomes are comprehensive and clinically reviewed by a respiratory clinician.
- Risk of bias will be formally assessed in a structured and standardised way using the Scottish Intercollegiate Guidelines Network (SIGN) methodology checklists.
- No geographic, time or language restrictions will be applied in an attempt to find all eligible studies.
- The search will be conducted in English language within English databases; thus we may miss relevant articles not captured by English language search engines.

For peer review only - http://bmjopen.bmj.com/site/about/guidelines.xhtml

3 Keywords

Cancer survivors, respiratory disorders, Covid-19, pneumonia, influenza, respiratory tract infections, asthma, COPD, interstitial lung disease, systematic review.

4 Introduction

Currently, 50% of people diagnosed with cancer are expected to survive for 10 or more years (1). The proportion is likely to increase further considering the rapid and continual improvements in cancer management and treatment. As more patients survive longer, they may experience additional health challenges during their survivorship. There is evidence that cancer survivors are at an increased risk of developing certain adverse outcomes, such as cardiovascular diseases (2) and mental health issues (3), that may cause morbidity and impaired quality of life. However, the long-term respiratory consequences of cancer and its treatment are less clear.

Respiratory diseases have an immense worldwide burden of morbidity and mortality, accounting for more than 10% of all disability-adjusted life-years (DALYs), second only to cardiovascular diseases (including stroke) (4). Furthermore, cancer can weaken the immune system which may increase the risk of respiratory infections (5). Additionally, cancer survivors often have a higher health burden. A quarter of cancer patients have pre-existing respiratory comorbidities (6) and there is concern that cancer and its treatment or shared risk factors may be conducive to worse outcomes than in the general population (7). Considering the high prevalence of respiratory disease in the general population, even a small increase in respiratory disease risk in cancer survivors will translate into an important impact on morbidity and mortality. Thus, identifying possible strategies to mitigate respiratory morbidity in cancer survivors would suppose an important improvement in absolute terms.

For peer review only - http://bmjopen.bmj.com/site/about/guidelines.xhtml

We aim to conduct a systematic review to summarise and assess the quality of the current knowledge from studies comparing survivors of adult cancer to individuals with no history of cancer for respiratory outcomes. We aim to evaluate relative risks of a broad spectrum of respiratory outcomes across a wide range of cancer types, in order to build a complete picture of the evidence on respiratory health during cancer survivorship.

Methods and analysis

This study protocol has been designed according to the Preferred Reporting Items for Systematic Review and Meta-Analysis Protocols (PRISMA-P), registered in the International Prospective Register of Ongoing Systematic Reviews (PROSPERO) and will be reported according to the PRISMA reporting guidelines. PROSPERO registration number: CRD42022311557.

4.1 Objectives

The specific objectives of this study are:

1. Summarise the current evidence on the association between cancer survivorship and incident respiratory disease outcomes. We will consider the relative risk of incident diagnosis, exacerbations (considered as GP visits or hospitalisations) and mortality outcomes separately for each respiratory disease of interest between cancer survivors and the cancer-free population.
2. Critically appraise the quality of the available evidence and evaluate the main sources of bias.
3. Identify evidence gaps and priorities for future research on respiratory health in cancer survivors.

For peer review only - http://bmjopen.bmj.com/site/about/guidelines.xhtml

4.2 Search strategy

The search will include articles published in Cochrane, Medline and Embase through to 08 March 2022. No geographic, time or language restrictions will be applied. We will use OVID© search interface to simultaneously search both Medline and Embase and we will conduct a separate search within Cochrane. The coverage of EMBASE spans from 1974,Medline from 1946 and Cochrane Reviews from 1996. We do not plan to include grey literature as epidemiological studies providing measures of effect with enough information to be assessable are likely to be published in academic journals. Furthermore, we do not plan to routinely contact all study authors. We will search title, abstract and subject headings using our created search expression. The search expression will include keywords and subject headings (both MeSH and Emtree) for the target population (cancer survivors) and the target outcomes (respiratory diseases). As an additional step to identify studies that may have been missed by the initial search strategy, we will examine the reference lists of all studies identified in the initial database search that meet the selection criteria. The list of selected respiratory outcomes and associated search terms were confirmed by a respiratory consultant (JQ). The search expression is included in Table 1

*Table 1. Search expression for both MEDLINE and EMBASE**

**Search expression**
1 *Cancer Survivors/ or exp cancer survivor
2 (cancer survivor* or "living with and beyond cancer" or "living with cancer").mp.
3 (respiratory and (outcome* or disease* or infection* or condition* or disorder or manifestation*)).mp.
4 (lung and (outcome* or disease* or infection* or condition* or disorder or manifestation*)).mp.
5 (pulmonary and (outcome* or disease* or infection* or condition* or disorder or manifestation*)).mp.
6 exp respiratory tract disease/ or exp lung disease/
7 ("upper respiratory infection*" or "upper respiratory tract infection*" or nasopharyngitis or rhinitis or pharyngitis or sinusitis or tonsilitis or laryngitis or tracheitis or laryngotracheitis).mp.
8 respiratory tract infections/ or common cold/ or empyema, pleural/ or laryngitis/ or pharyngitis/ or rhinitis/ or sinusitis/ or whooping cough/ or exp upper respiratory tract/ or exp upper respiratory tract congestion/ or exp upper respiratory tract infection/
9 bronchitis.mp. or Bronchitis/ or Infectious bronchitis virus/

For peer review only - http://bmjopen.bmj.com/site/about/guidelines.xhtml

10 pulmonary diseases/ or chronic obstructive/ or bronchitis, chronic/ or pulmonary emphysema/ or Bronchitis, Chronic/ or exp chronic bronchitis/ or exp bronchitis/
11 (influenza* or flu).mp. or influenza, human/
12 exp influenza/
13 Pneumonia/ or pneumonia.mp.
14 exp pneumonia/
15 Asthma/ or asthma.mp.
16 exp asthma/
17 (chronic obstructive pulmonary disease or COPD or chronic bronchitis or emphysema).mp.
18 exp chronic obstructive lung disease/
19 interstitial lungs disease.mp. or Lung Diseases, Interstitial/
20 exp interstitial lung disease/
21 Pulmonary Fibrosis/
22 (pulmonary fibrosis or lung fibrosis).mp.
23 exp lung fibrosis/
24 pneumonitis.mp.
25 exp coronavirus disease 2019/ or exp COVID-19/ or (Covid-19 or SARS-CoV-2 or coronavirus 19).mp.
26 3 or 4 or 5 or 6 or 7 or 8 or 9 or 10 or 11 or 12 or 13 or 14 or 15 or 16 or 17 or 18 or 19 or 20 or 21 or 22 or 23 or 24 or 25
27 1 or 2
32 26 and 27

*Both MeSH terms and Emtree terms were included in our search expression.

We defined respiratory disease as any upper or lower respiratory condition. See Table 2 for list of included respiratory diseases.

Table 2. Categorisation of respiratory diseases included in the systematic literature review

Category	Conditions of interest
Covid-19	Infection with SARS-CoV-19
Upper respiratory infections	Nasopharyngitis, rhinitis, pharyngitis, sinusitis, tonsilitis, laryngitis, tracheitis, laryngotracheitis
Bronchitis	Acute Bronchitis
Influenza	Influenza
Pneumonia	Pneumonia
Asthma	Asthma
COPD	chronic obstructive pulmonary disease, chronic bronchitis, emphysema
Pneumonitis	pneumonitis
Fibrosis	pulmonary fibrosis
Interstitial lung disease	Interstitial lung disease

4.3 Screening

The screening will be conducted in two stages:

For peer review only - http://bmjopen.bmj.com/site/about/guidelines.xhtml

1) Screening of titles and abstracts: Titles and abstracts will be screened against the selection criteria by one reviewer. A second reviewer will screen a random sample of ten percent of articles identified in the initial search. Any discrepancies will be resolved by discussion between the reviewers and the wider study team. Studies which meet all the inclusion criteria and none of the exclusion criteria (see below) will be considered for full text review. If the study cannot be unequivocally excluded during this phase, it will be considered for full text review.
2) Full text review: This step will be conducted independently by two reviewers in full. Any discrepancies will be resolved by discussion between the reviewers and the wider study team.

We will use the reference manager Endnote 20 to store citations and screen all identified references.

4.4 Selection criteria

The inclusion criteria for the systematic review will be:

1) The exposure is cancer survivorship. In this review our focus will be on the survivorship period after initial active cancer treatment, so the exposed group must include follow-up time beyond the period of active treatment or the first year since diagnosis.
2) The focus is on adult cancer survivors; thus we will only include studies where individuals were aged ≥18 years at diagnosis.
3) The study evaluates at least one respiratory disease of interest as an outcome of interest (See Table 2 for details).
4) The study involves a comparator cohort of cancer-free individuals or a general population estimate used to calculate indirect measures such as Standardised Mortality Ratios. No other criteria will be set for the comparator.
5) The study has an observational longitudinal cohort, matched cohort or case-control design.

For peer review only

6) The study provides a relative measure of effect for a respiratory disease of interest in individuals with cancer compared to cancer-free controls. Examples of relevant measures of effect are relative risk ratios, odds ratios, rate ratios, and hazard ratios. If studies provide sufficient information on measures of frequency for any of the respiratory diseases of interest measured in individuals with cancer and cancer-free controls to allow the calculation measure of effect between the groups, the study will also be included.

We will exclude:

1) Duplicate studies, defined as repeated articles, using the remove duplicate function of the software prior to conducting the manual screening. Any articles not identified by the software, will be removed manually by the reviewers.
2) Studies where the exposed group is exclusively composed of cancer patients under active treatment as well as cancer cohorts whose average follow-up time is ≤1 year.
3) Studies in which the exposed group includes individuals without cancer (e.g., families and caregivers of the patient).
4) Intervention studies, cross-sectional studies and studies that do not use original data (such as review articles, comments, letters, editorials and protocols). Any review study will be excluded but flagged and the citation lists will be evaluated in order to identify potentially eligible studies that were not captured in our search.
5) Conference abstracts will be excluded if no corresponding full-text journal article is found as we consider that the information in an abstract will not be sufficient to complete our data extraction nor conduct an accurate bias assessment.

While articles will not be excluded on the basis of language, practical limitations mean that we will only evaluate those studies captured by an English search strategy. Articles in languages not spoken by the study team will be translated using translation software. We will exclude full-text articles which are written in non-roman alphabets due to the limitations of our translation software.

For peer review only

For peer review only - http://bmjopen.bmj.com/site/about/guidelines.xhtml

Reasons for exclusion will be collected in a cascade manner, meaning that papers that fail to meet any of the selection criteria will be automatically excluded and not further assessed. Where multiple papers are found to report data conducted on the same study population sample, these will be flagged, but not excluded. The results of these studies cannot be considered independent and thus the inclusion and/or interpretation of these studies will be considered on a case-by-case basis after discussion within the study team. Any inclusion or exclusions of studies of this type will be detailed in the data extraction phase and reported in the results section of the report.

4.5 Data extraction

Data will initially be extracted from included studies into a spreadsheet. Data extraction will commence in July 2022. At minimum, we will detail information on the study article, population sample description, study design, exposure, comparator and outcome definitions, follow-up, measures of effect, measures of frequency and statistical methodology. The exact data items extracted are listed in Table 3.

Table 3. Data items for extraction

Data items:
Administrative:
• Authors<br>• Journal<br>• Year of publication<br>• Financial/funding sources<br>• Conflict of interest reported
Data/design/population:
• Country/countries of origin<br>• Data source<br>• Time period covered<br>• Study setting<br>• Study design<br>• Sample size
Comparison group:
• Selection criteria of the comparison group<br>• Mean age of the comparison group
Exposure:

For peer review only

• Selection criteria for exposed group<br>• Definition of cancer survivorship<br>• Time since cancer diagnosis (summary stats)<br>• Cancer Stage (summary stats)
Outcome:
• Respiratory disease category<br>• Respiratory disease definition<br>• Duration of follow-up (if applicable)
Statistical methods:
• Statistical method for the calculation of estimates<br>• Statistical method for the calculation of the method of precision<br>• List of confounders included in the adjusted analysis (if applicable)
Results
• Measure of frequency + precision estimate (comparison group)<br>• Measure of frequency + precision estimate (exposed group)<br>• Measure of effect + precision estimate (crude)<br>• Measure of effect + precision estimate (adjusted)

When multiple *different* respiratory disease conditions or cancer types are reported in the same study, we will record or calculate information for all cancer types and respiratory outcomes. When multiple estimates are reported for the *same condition* (e.g., if probable and confirmed influenza were reported as separate outcomes) we will only record or calculate the measure of effect for the outcome corresponding to the stronger certainty of diagnosis (in our illustrative example, we would select confirmed influenza). For *each* outcome we will record both the crude and adjusted effect measures as well as the list of confounders included in the adjusted analysis. If the results are stratified by severity, we will record all severity categories for said outcome.

4.6 Risk of bias assessment

We will use the Scottish Intercollegiate Guidelines Network (SIGN) methodology checklists as guidance to assess the risk of bias for each study design. The checklists are provided in Appendix 1. In brief, we will assess if the study has a focussed aim, and whether there were opportunities for bias in in the definition of the exposures and outcomes, selection of the study population and comparator group, and potential differential losses during follow up. We will also assess how each study addresses confounding.

For peer review only

For peer review only - http://bmjopen.bmj.com/site/about/guidelines.xhtml

4.7 Data Analysis

We will initially present the key characteristics and results of all studies in descriptive tables. We will stratify the presentation of results by likely sources of heterogeneity such as cancer type and respiratory disease category (See Table 2). If sufficient studies are found, we will also stratify by different population definitions (i.e., if we find multiple definitions for cancer survivorship). We will meta-analyse groups of studies if we find sufficient studies that are sufficiently homogeneous in terms of population and outcomes for which the calculation of a summary measure would be meaningful. In the case that a summary estimate is calculated, we will use random-effects model. We will explore the consistency between studies by using the I^2 statistic.

4.8 Patient and public involvement

No patients were involved in the development of this protocol

5 Ethics and dissemination

To our knowledge, no comparable review has previously been conducted on respiratory outcomes in survivors of adult cancer. We aim to identify all available literature on a wide range of respiratory outcomes and cancer types considered, with no language, geographical or date limitations. This will provide a comprehensive picture of the spectrum of respiratory conditions that affect cancer survivors as well as understand if cancer-specific or respiratory condition-specific associations have been noted in the literature. Considering the high prevalence of respiratory disease in the general population, even a small increase in respiratory disease risk in cancer survivors will translate into an important impact on morbidity and mortality. The results of this review can be used to better understand respiratory morbidity in cancer survivors, identify evidence gaps and priorities for future research, and help devise possible mitigation strategies. Results will be disseminated to clinical audiences and may support clinicians and decision-makers in developing survivorship care guidelines.

For peer review only - http://bmjopen.bmj.com/site/about/guidelines.xhtml

While this protocol has been developed according to the PRISMA-P guidelines and has been registered on PROSPERO (ref: PROSPERO CRD42022311557), some potential limitations are worth noting. Firstly, there is currently no universally accepted definition of cancer survivorship (8), and this review focusses on the late respiratory challenges of cancer and its treatment. Consequentially, we have adopted the European Organisation for Research and Treatment of Cancer (EORTC) definition which defines cancer survivor as "individuals diagnosed with cancer who have completed their primary treatment (maintenance treatment can be ongoing)"(9). We have used an inclusive approach towards this definition and will consider that the period of active treatment is expected to have been completed after 1 year since diagnosis, thus we will not exclude studies that include patients from cancer diagnosis with a mean follow-up of <1 year. This is in line with the International Agency for Research on Cancer (IARC) categorisation of net survival: 1 year survival (initial treatment), 3 year survival (clinical follow-up) and five year survival (cure) (10).

Studies comparing long-term outcomes in cancer survivors and controls may be subject to a number of biases including selection bias, detection biases, and competing-risks. We will evaluate the risk of bias in each included study in order to aid the interpretation of results, though we acknowledge that not all types of bias are specifically addressed by the SIGN checklists. Our review will also have other limitations. We may miss some relevant studies due to an imperfect search strategy, though hand searching of reference lists should identify additional papers and allow us to refine our search terms if needed. Finally, we are not including conference abstracts or preprints which could potentially exclude some relevant results.

Considering the high prevalence of respiratory disease in the general population, even a small increase in respiratory disease risk in cancer survivors will translate into an important impact on morbidity and mortality. The results of this review can be used to better understand respiratory morbidity in cancer survivors, identify evidence gaps and priorities for future research, and help devise possible mitigation strategies.

For peer review only - http://bmjopen.bmj.com/site/about/guidelines.xhtml

6 Abbreviation list

EORTC	European Organisation for Research and Treatment of Cancer
IARC	International Agency for Research on Cancer
PRISMA	Preferred reporting items for systematic review and meta-analysis
PRISMA – P	Preferred reporting items for systematic review and meta-analysis protocol
PROSPERO	International Prospective Register of Ongoing Systematic Reviews
SIGN	Scottish Intercollegiate Guidelines Network

7 Conflict of interests

No competing interests to declare

8 Author Statement

KB, HC and KA designed the study. KA wrote the first draft of the protocol. JQ provided clinical input for the selection of the study outcomes. All authors made substantial contributions to the manuscript, revised it critically for important intellectual content and read and approved the final version. KA is the guarantor of the review.

9 Data statement

The protocol is registered on PROSPERO (registration number: CRD42022311557). The data extraction sheet will be provided as an appendix to any published article

10 Funding

KA is supported by the Medical Research Council Intercollegiate Doctoral Training Partnership Studentship (Grant no MR/N013638/1). KB is funded by a Wellcome Senior Research Fellowship (grant number 220283/z/20/z). The funders had no role in developing this protocol.

11 Acknowledgements

We thank Ruchika Jain for agreeing to assist in conducting the screening of full-text articles.

For peer review only

For peer review only - http://bmjopen.bmj.com/site/about/guidelines.xhtml

12 References

1. Cancer Research UK. Cancer survival statistics for all cancers combined [Available from: https://www.cancerresearchuk.org/health-professional/cancer-statistics/survival/all-cancers-combined.

2. Strongman H, Gadd S, Matthews A, Mansfield KE, Stanway S, Lyon AR, et al. Medium and long-term risks of specific cardiovascular diseases in survivors of 20 adult cancers: a population-based cohort study using multiple linked UK electronic health records databases. Lancet (London, England). 2019;394(10203):1041-54.

3. Carreira H, Williams R, Dempsey H, Stanway S, Smeeth L, Bhaskaran K. Quality of life and mental health in breast cancer survivors compared with non-cancer controls: a study of patient-reported outcomes in the United Kingdom. J Cancer Surviv. 2021;15(4):564-75.

4. Wang H, Naghavi M, Allen C, Barber RM, Bhutta ZA, Carter A, et al. Global, regional, and national life expectancy, all-cause mortality, and cause-specific mortality for 249 causes of death, 1980–2015: a systematic analysis for the Global Burden of Disease Study 2015. The Lancet. 2016;388(10053):1459-544.

5. Kang DH, Weaver MT, Park NJ, Smith B, McArdle T, Carpenter J. Significant impairment in immune recovery after cancer treatment. Nurs Res. 2009;58(2):105-14.

6. Ogle KS, Swanson GM, Woods N, Azzouz F. Cancer and comorbidity. Cancer. 2000;88(3):653-63.

7. Carreira H, Strongman H, dos-Santos-Silva I, Smeeth L, Bhaskaran K, Peppa M, et al. Prevalence of COVID-19-related risk factors and risk of severe influenza outcomes in cancer

For peer review only - http://bmjopen.bmj.com/site/about/guidelines.xhtml

survivors: A matched cohort study using linked English electronic health records data. EClinicalMedicine. 2020;29-30:100656.
8. Dirven L, van de Poll-Franse LV, Aaronson NK, Reijneveld JC. Controversies in defining cancer survivorship. The Lancet Oncology. 2015;16(6):610-2.
9. Moser EC, Meunier F. Cancer survivorship: A positive side-effect of more successful cancer treatment. European Journal of Cancer Supplements. 2014;12(1):1-4.
10. Arnold M RM, Lam F, Bray F, Ervik M, Soerjomataram I (2019). . accessed [day/month/year]. ICBP SURVMARK-2 online tool: International Cancer Survival Benchmarking. Lyon, France: International Agency for Research on Cancer Available from: http://gco.iarc.fr/survival/survmark, 2019 [

For peer review only

For peer review only - http://bmjopen.bmj.com/site/about/guidelines.xhtml

Table A 1. Methodology checklist case control studies

Methodology Checklist 4: Case-control studies

SIGN

Study identification (*Include author, title, year of publication, journal title, pages*)

Guideline topic:	Key Question No:	Reviewer:

Before completing this checklist, consider:

1. Is the paper really a case-control study? If in doubt, check the study design algorithm available from SIGN and make sure you have the correct checklist.
2. Is the paper relevant to key question? Analyse using PICO (Patient or Population Intervention Comparison Outcome). IF NO REJECT (give reason below). IF YES complete the checklist.

Reason for rejection: Reason for rejection: 1. Paper not relevant to key question ☐ 2. Other reason ☐ (please specify):

SECTION 1: INTERNAL VALIDITY

	In an well conducted case control study:	***Does this study do it?***
1.1	The study addresses an appropriate and clearly focused question.[i]	Yes ☐ No ☐ Can't say ☐
SELECTION OF SUBJECTS		
1.2	The cases and controls are taken from comparable populations.[ii]	Yes ☐ No ☐ Can't say ☐
1.3	The same exclusion criteria are used for both cases and controls.[iii]	Yes ☐ No ☐ Can't say ☐
1.4	What percentage of each group (cases and controls) participated in the study?[iv]	Cases: Controls:
1.5	Comparison is made between participants and non-participants to establish their similarities or differences.[v]	Yes ☐ No ☐ Can't say ☐
1.6	Cases are clearly defined and differentiated from controls.[vi]	Yes ☐ No ☐ Can't say ☐
1.7	It is clearly established that controls are non-cases.[vii]	Yes ☐ No ☐ Can't say ☐
ASSESSMENT		

For peer review only

1.8	Measures will have been taken to prevent knowledge of primary exposure influencing case ascertainment.[viii]	Yes ☐ No ☐ Can't say ☐ Does not apply ☐
1.9	Exposure status is measured in a standard, valid and reliable way.[ix]	Yes ☐ No ☐ Can't say ☐
CONFOUNDING		
1.10	The main potential confounders are identified and taken into account in the design and analysis.[x]	Yes ☐ No ☐ Can't say ☐
STATISTICAL ANALYSIS		
1.11	Confidence intervals are provided.[xi]	Yes ☐ No ☐
**SECTION 2: OVERALL ASSESSMENT OF THE STUDY**		
2.1	How well was the study done to minimise the risk of bias or confounding? [xii]	High quality (++) ☐ Acceptable (+) ☐ Unacceptable – reject 0 ☐
2.2	Taking into account clinical considerations, your evaluation of the methodology used, and the statistical power of the study, do you think there is clear evidence of an association between exposure and outcome?	Yes ☐ Can't say ☐ No ☐
2.3	Are the results of this study directly applicable to the patient group targeted by this guideline?	Yes ☐ No ☐
2.4	**Notes.** Summarise the authors conclusions. Add any comments on your own assessment of the study, and the extent to which it answers your question and mention any areas of uncertainty raised above..	

For peer review only

[i] Unless a clear and well defined question is specified in the report of the review, it will be difficult to assess how well it has met its objectives or how relevant it is to the question you are trying to answer on the basis of the conclusions.

[ii] Study participants may be selected from the target population (all individuals to which the results of the study could be applied), the source population (a defined subset of the target population from which participants are selected), or from a pool of eligible subjects (a clearly defined and counted group selected from the source population. **If the study does not include clear definitions of the source population it should be rejected.**

[iii] All selection and exclusion criteria should be applied equally to cases and controls. Failure to do so may introduce a significant degree of bias into the results of the study.

[iv] Differences between the eligible population and the participants are important, as they may influence the validity of the study. A participation rate can be calculated by dividing the number of study participants by the number of eligible subjects. It is more useful if calculated separately for cases and controls. If the participation rate is low, or there is a large difference between the two groups, the study results may well be invalid due to differences between participants and non-participants. In these circumstances, the study should be downgraded, and rejected if the differences are very large.

[v] Even if participation rates are comparable and acceptable, it is still possible that the participants selected to act as cases or controls may differ from other members of the source population in some significant way. A well conducted case-control study will look at samples of the non-participants among the source population to ensure that the participants are a truly representative sample.

[vi] The method of selection of cases is of critical importance to the validity of the study. Investigators have to be certain that cases are truly cases, but must balance this with the need to ensure that the cases admitted into the study are representative of the eligible population. **The issues involved in case selection are complex, and should ideally be evaluated by someone with a good understanding of the design of case-control studies. If the study does not comment on how cases were selected, it is probably safest to reject it as a source of evidence.**

[vii] Just as it is important to be sure that cases are true cases, it is important to be sure that controls do not have the outcome under investigation. Control subjects should be chosen so that information on exposure status can be obtained or assessed in a similar way to that used for the selection of cases. If the methods of control selection are not described, the study should be rejected. **If different methods of selection are used for cases and controls the study should be evaluated by someone with a good understanding of the design of case-control studies.**

[viii] If there is a possibility that case ascertainment can be influenced by knowledge of exposure status, assessment of any association is likely to be biased. A well conducted study should take this into account in the design of the study.

[ix] The primary outcome measures used should be clearly stated in the study. **If the outcome measures are not stated, or the study bases its main conclusions on secondary outcomes, the study should be rejected.** Where outcome measures require any degree of subjectivity, some evidence should be provided that the measures used are reliable and have been validated prior to their use in the study.

[x] Confounding is the distortion of a link between exposure and outcome by another factor that is associated with both exposure and outcome. The possible presence of confounding factors is one of the principal reasons why observational studies are not more highly rated as a source of evidence. The study should indicate which potential confounders have been considered, and how they have been allowed for in the analysis. Clinical judgement should be applied to consider whether all likely confounders have been considered. If the measures used to address confounding are considered inadequate, the study should be downgraded or rejected. **A study that does not address the possibility of confounding should be rejected.**

[xi] Confidence limits are the preferred method for indicating the precision of statistical results, and can be used to differentiate between an inconclusive study and a study that shows no effect. Studies that report a single value with no assessment of precision should be treated with extreme caution.

[xii] Rate the overall methodological quality of the study, using the following as a guide: **High quality** (++): Majority of criteria met. Little or no risk of bias. Results unlikely to be changed by further research. **Acceptable** (+): Most criteria met. Some flaws in the study with an associated risk of bias, Conclusions may change in the light of further studies. **Low quality** (0): Either most criteria not met, or significant flaws relating to key aspects of study design. Conclusions likely to change in the light of further studies.

For peer review only

For peer review only

Table A2, Methodology checklist cohort studies

SIGN

Methodology Checklist 3: Cohort studies

Study identification (*Include author, title, year of publication, journal title, pages*)

Guideline topic:	Key Question No:	Reviewer:

Before completing this checklist, consider:

1. Is the paper really a cohort study? If in doubt, check the study design algorithm available from SIGN and make sure you have the correct checklist.
2. Is the paper relevant to key question? Analyse using PICO (Patient or Population Intervention Comparison Outcome). IF NO REJECT (give reason below). IF YES complete the checklist..

Reason for rejection: 1. Paper not relevant to key question ☐ 2. Other reason ☐ (please specify):

Please note that a retrospective study (ie a database or chart study) cannot be rated higher than +.

SECTION 1: INTERNAL VALIDITY

	In a well conducted cohort study:	***Does this study do it?***
1.1	The study addresses an appropriate and clearly focused question.[xii]	Yes □ No □<br>Can't say □
**SELECTION OF SUBJECTS**		
1.2	The two groups being studied are selected from source populations that are comparable in all respects other than the factor under investigation.[xii]	Yes □ No □<br>Can't say □ Does not apply □
1.3	The study indicates how many of the people asked to take part did so, in each of the groups being studied.[xii]	Yes □ No □<br>Does not apply □
1.4	The likelihood that some eligible subjects might have the outcome at the time of enrolment is assessed and taken into account in the analysis.[xii]	Yes □ No □<br>Can't say □ Does not apply □
1.5	What percentage of individuals or clusters recruited into each arm of the study dropped out before the study was completed.[xii]	
1.6	Comparison is made between full participants and those lost to follow up, by exposure status.[xii]	Yes □ No □<br>Can't say □ Does not apply □

ASSESSMENT

For peer review only - http://bmjopen.bmj.com/site/about/guidelines.xhtml

1.7	The outcomes are clearly defined.[xii]	Yes □ Can't say □	No □
1.8	The assessment of outcome is made blind to exposure status. If the study is retrospective this may not be applicable.[xii]	Yes □ Can't say □	No □ Does not apply □
1.9	Where blinding was not possible, there is some recognition that knowledge of exposure status could have influenced the assessment of outcome.[xii]	Yes □ Can't say □	No □ □
1.10	The method of assessment of exposure is reliable.[xii]	Yes □ Can't say □	No □
1.11	Evidence from other sources is used to demonstrate that the method of outcome assessment is valid and reliable.[xii]	Yes □ Can't say □	No □ Does not apply□
1.12	Exposure level or prognostic factor is assessed more than once.[xii]	Yes □ Can't say □	No □ Does not apply □
CONFOUNDING			
1.13	The main potential confounders are identified and taken into account in the design and analysis.[xii]	Yes □ Can't say □	No □
STATISTICAL ANALYSIS			
1.14	Have confidence intervals been provided?[xii]	Yes □	No □
**SECTION 2: OVERALL ASSESSMENT OF THE STUDY**			
2.1	How well was the study done to minimise the risk of bias or confounding?[xii]	High quality (++) □ Acceptable (+) □ Unacceptable – reject 0	
2.2	Taking into account clinical considerations, your evaluation of the methodology used, and the statistical power of the study, do you think there is clear evidence of an association between exposure and outcome?	Yes ☐ Can't say ☐	No ☐
2.3	Are the results of this study directly applicable to the patient group targeted in this guideline?	Yes □	No □
2.4	**Notes.** Summarise the authors conclusions. Add any comments on your own assessment of the study, and the extent to which it answers your question and mention any areas of uncertainty raised above.		

For peer review only

[xii] Unless a clear and well defined question is specified in the report of the review, it will be difficult to assess how well it has met its objectives or how relevant it is to the question you are trying to answer on the basis of the conclusions.

[xii] This relates to **selection bias**.* It is important that the two groups selected for comparison are as similar as possible in all characteristics except for their exposure status, or the presence of specific prognostic factors or prognostic markers relevant to the study in question.

[xii] This relates to **selection bias**.* The participation rate is defined as the number of study participants divided by the number of eligible subjects, and should be calculated separately for each branch of the study. A large difference in participation rate between the two arms of the study indicates that a significant degree of **selection bias*** may be present, and the study results should be treated with considerable caution.

[xii] If some of the eligible subjects, particularly those in the unexposed group, already have the outcome at the start of the trial the final result will be subject to **performance bias.*** A well conducted study will attempt to estimate the likelihood of this occurring, and take it into account in the analysis through the use of sensitivity studies or other methods.

[xii] This question relates to the risk of **attrition bias**.*The number of patients that drop out of a study should give concern if the number is very high. Conventionally, a 20% drop out rate is regarded as acceptable, but in observational studies conducted over a lengthy period of time a higher drop out rate is to be expected. A decision on whether to downgrade or reject a study because of a high drop out rate is a matter of judgement based on the reasons why people dropped out, and whether drop out rates were comparable in the exposed and unexposed groups. Reporting of efforts to follow up participants that dropped out may be regarded as an indicator of a well conducted study.

[xii] For valid study results, it is essential that the study participants are truly representative of the source population. It is always possible that participants who dropped out of the study will differ in some significant way from those who remained part of the study throughout. A well conducted study will attempt to identify any such differences between full and partial participants in both the exposed and unexposed groups. This relates to the risk of **attrition bias**.* Any unexplained differences should lead to the study results being treated with caution.

[xii] This relates to the risk of **detection bias**.* Once enrolled in the study, participants should be followed until specified end points or outcomes are reached. In a study of the effect of exercise on the death rates from heart disease in middle aged men, for example, participants might be followed up until death, or until reaching a predefined age. **If outcomes and the criteria used for measuring them are not clearly defined, the study should be rejected.**

[xii] This relates to the risk of **detection bias.*** If the assessor is blinded to which participants received the exposure, and which did not, the prospects of unbiased results are significantly increased. Studies in which this is done should be rated more highly than those where it is not done, or not done adequately.

[xii] This relates to the risk of **detection bias.*** Blinding is not possible in many cohort studies. In order to asses the extent of any bias that may be present, it may be helpful to compare process measures used on the participant groups - e.g. frequency of observations, who carried out the observations, the degree of detail and completeness of observations. If these process measures are comparable between the groups, the results may be regarded with more confidence.

[xii] This relates to the risk of **detection bias.*** A well conducted study should indicate how the degree of exposure or presence of prognostic factors or markers was assessed. Whatever measures are used must be sufficient to establish clearly that participants have or have not received the exposure under investigation and the extent of such exposure, or that they do or do not possess a particular prognostic marker or factor. Clearly described, reliable measures should increase the confidence in the quality of the study

[xii] This relates to the risk of **detection bias.*** The primary outcome measures used should be clearly stated in the study. **If the outcome measures are not stated, or the study bases its main conclusions on secondary outcomes, the study should be rejected.** Where outcome measures require any degree of subjectivity, some evidence should be provided that the measures used are reliable and have been validated prior to their use in the study.

[xii] This relates to the risk of **detection bias.*** Confidence in data quality should be increased if exposure level is measured more than once in the course of the study. Independent assessment by more than one investigator is preferable.

[xii] Confounding is the distortion of a link between exposure and outcome by another factor that is associated with both exposure and outcome. The possible presence of confounding factors is one of the principal reasons why observational studies are not more highly rated as a source of evidence. The report of the study should indicate which potential confounders have been considered, and how they have been assessed or allowed for in the analysis. Clinical judgement should be applied to consider whether all likely confounders have been considered. If the measures used to address confounding are considered inadequate, the study should be downgraded or rejected, depending on how serious the risk

For peer review only

of confounding is considered to be. **A study that does not address the possibility of confounding should be rejected.**

[xii] Confidence limits are the preferred method for indicating the precision of statistical results, and can be used to differentiate between an inconclusive study and a study that shows no effect. Studies that report a single value with no assessment of precision should be treated with extreme caution.

[xii] Rate the overall methodological quality of the study, using the following as a guide: **High quality** (++): Majority of criteria met. Little or no risk of bias. Results unlikely to be changed by further research. **Acceptable** (+): Most criteria met. Some flaws in the study with an associated risk of bias, Conclusions may change in the light of further studies. **Low quality** (0): Either most criteria not met, or significant flaws relating to key aspects of study design. Conclusions likely to change in the light of further studies.

For peer review only

PRISMA-P 2015 Checklist

This checklist has been adapted for use with systematic review protocol submissions to BioMed Central journals from Table 3 in Moher D et al: Preferred reporting items for systematic review and meta-analysis protocols (PRISMA-P) 2015 statement. *Systematic Reviews* 2015 **4**:1

An Editorial from the Editors-in-Chief of *Systematic Reviews* details why this checklist was adapted - Moher D, Stewart L & Shekelle P: Implementing PRISMA-P: recommendations for prospective authors. *Systematic Reviews* 2016 **5**:15

Section/topic	#	Checklist item	Information reported		Line number(s)
			Yes	No	
**ADMINISTRATIVE INFORMATION**					
**Title**					
Identification	1a	Identify the report as a protocol of a systematic review	☒	☐	Line 1-2
Update	1b	If the protocol is for an update of a previous systematic review, identify as such	☐	☒	Not applicable
**Registration**	2	If registered, provide the name of the registry (e.g., PROSPERO) and registration number in the Abstract	☒	☐	Line 38 & 79
**Authors**					
Contact	3a	Provide name, institutional affiliation, and e-mail address of all protocol authors; provide physical mailing address of corresponding author	☒	☐	Line 4-13
Contributions	3b	Describe contributions of protocol authors and identify the guarantor of the review	☒	☐	Line 245 - 249
**Amendments**	4	If the protocol represents an amendment of a previously completed or published protocol, identify as such and list changes; otherwise, state plan for documenting important protocol amendments	☐	☒	Not applicable
**Support**					
Sources	5a	Indicate sources of financial or other support for the review	☒	☐	Line 253-256
Sponsor	5b	Provide name for the review funder and/or sponsor	☒	☐	Line 253-256
Role of sponsor/funder	5c	Describe roles of funder(s), sponsor(s), and/or institution(s), if any, in developing the protocol	☒	☐	Line 253-256
**INTRODUCTION**					
**Rationale**	6	Describe the rationale for the review in the context of what is already known	☒	☐	Line 53-73

For peer review only

BioMed Central The Open Access Publisher

For peer review only - http://bmjopen.bmj.com/site/about/guidelines.xhtml

Section/topic	#	Checklist item	Information reported Yes	Information reported No	Line number(s)
**Objectives**	7	Provide an explicit statement of the question(s) the review will address with reference to participants, interventions, comparators, and outcomes (PICO)	☒	☐	Line 80-90
**METHODS**					
**Eligibility criteria**	8	Specify the study characteristics (e.g., PICO, study design, setting, time frame) and report characteristics (e.g., years considered, language, publication status) to be used as criteria for eligibility for the review	☒	☐	Line 123-168
**Information sources**	9	Describe all intended information sources (e.g., electronic databases, contact with study authors, trial registers, or other grey literature sources) with planned dates of coverage	☒	☐	Line 91-105
**Search strategy**	10	Present draft of search strategy to be used for at least one electronic database, including planned limits, such that it could be repeated	☒	☐	Line 106
STUDY RECORDS					
Data management	11a	Describe the mechanism(s) that will be used to manage records and data throughout the review	☒	☐	Line 123
Selection process	11b	State the process that will be used for selecting studies (e.g., two independent reviewers) through each phase of the review (i.e., screening, eligibility, and inclusion in meta-analysis)	☒	☐	Line 111- 122
Data collection process	11c	Describe planned method of extracting data from reports (e.g., piloting forms, done independently, in duplicate), any processes for obtaining and confirming data from investigators	☒	☐	Line 169 - 191
**Data items**	12	List and define all variables for which data will be sought (e.g., PICO items, funding sources), any pre-planned data assumptions and simplifications	☒	☐	Line 175
**Outcomes and prioritization**	13	List and define all outcomes for which data will be sought, including prioritization of main and additional outcomes, with rationale	☒	☐	Line 110 & Line 177-184
**Risk of bias in individual studies**	14	Describe anticipated methods for assessing risk of bias of individual studies, including whether this will be done at the outcome or study level, or both; state how this information will be used in data synthesis	☒	☐	Line 185 - 191
DATA					
**Synthesis**	15a	Describe criteria under which study data will be quantitatively synthesized	☒	☐	Line 180-181
	15b	If data are appropriate for quantitative synthesis, describe planned summary measures, methods of handling data, and methods of combining data from studies, including any planned exploration of consistency (e.g., I^2, Kendall's tau)	☒	☐	Line 192 - 200
	15c	Describe any proposed additional analyses (e.g., sensitivity or subgroup analyses, meta-	☒	☐	Line 192 - 200

For peer review only

BioMed Central The Open Access Publisher

Section/topic	#	Checklist item	Information reported: Yes	Information reported: No	Line number(s)
		regression)			
	15d	If quantitative synthesis is not appropriate, describe the type of summary planned	☒	☐	Line 192 - 200
**Meta-bias(es)**	16	Specify any planned assessment of meta-bias(es) (e.g., publication bias across studies, selective reporting within studies)	☐	☒	Not applicable
**Confidence in cumulative evidence**	17	Describe how the strength of the body of evidence will be assessed (e.g., GRADE)	☐	☒	Not applicable

For peer review only

BioMed Central
The Open Access Publisher
